# Spontaneously coherent orbital coupling of counterrotating exciton polaritons in annular perovskite microcavities

**DOI:** 10.1038/s41377-021-00478-w

**Published:** 2021-03-01

**Authors:** Jun Wang, Huawen Xu, Rui Su, Yutian Peng, Jinqi Wu, Timothy C. H. Liew, Qihua Xiong

**Affiliations:** 1 Division of Physics and Applied Physics, School of Physical and Mathematical Sciences, Nanyang Technological University, Singapore, Singapore; 2 State Key Laboratory of Low-Dimensional Quantum Physics and Department of Physics, Tsinghua University, Beijing, China; 3 MajuLab, International Joint Research Unit UMI 3654, CNRS, Université Côte d’Azur, Sorbonne Université, National University of Singapore, Nanyang Technological University, Singapore, Singapore; 4 Beijing Academy of Quantum Information Sciences, Beijing, 100193 P.R. China

**Keywords:** Polaritons, Photonic devices

## Abstract

Exciton-polariton condensation is regarded as a spontaneous macroscopic quantum phenomenon with phase ordering and collective coherence. By engineering artificial annular potential landscapes in halide perovskite semiconductor microcavities, we experimentally and theoretically demonstrate the room-temperature spontaneous formation of a coherent superposition of exciton-polariton orbital states with symmetric petal-shaped patterns in real space, resulting from symmetry breaking due to the anisotropic effective potential of the birefringent perovskite crystals. The lobe numbers of such petal-shaped polariton condensates can be precisely controlled by tuning the annular potential geometry. These petal-shaped condensates form in multiple orbital states, carrying locked alternating π phase shifts and vortex–antivortex superposition cores, arising from the coupling of counterrotating exciton-polaritons in the confined circular waveguide. Our geometrically patterned microcavity exhibits promise for realizing room-temperature topological polaritonic devices and optical polaritonic switches based on periodic annular potentials.

## Introduction

Self-organizing pattern formation phenomena frequently emerge in various interdisciplinary research fields^1,2^, such as self-assembly of molecules^3^ and liquid crystal ordering in chemistry^4^. In particular, closed-loop lasing systems with imposed cylindrical symmetry^5–7^ exhibit spontaneous pattern formation due to the coupling of the rotation around a closed ring to the phase of a quantum wavefunction. Periodic boundary conditions with circular symmetry can establish some phase ordering and phase locking of the quantum wavefunction at macroscopic scales, giving rise to spatially ordered patterns in the density distribution. This scheme, assisted by geometric symmetry, helps to simulate the arrangement and spontaneous ordering of spins in condensed matter physics to facilitate the study of the orbital angular momentum (OAM) of light^8–11^.

Exciton-polaritons (EPs) are bosonic quasiparticles generated from the quantum hybridization of excitons and confined photons. Due to their strong nonlinearity and low effective mass, EPs can achieve Bose–Einstein condensation at much higher temperatures than is possible in ultracold atomic systems^12^, forming long-range coherence in a macroscale region^13,14^. Meanwhile, the process of condensation through stimulated scattering exhibits non-equilibrium characteristics, complying with the competition between loss and gain^15^. This driven-dissipative mechanism enables EPs to condense into macroscopically coherent many-body states^16–18^, which allows the spontaneous pattern formation of phase-locked polariton condensates to emulate 1D ordered spin chains by means of the phase degree of freedom^19–21^. Concurrently, the localization of polaritons via the confinement of the photonic components and the sculpting of the microstructure in a planar microcavity enables precise manipulations of not only the intersite coupling of polariton wavefunctions^22,23^, but also the OAM^24,25^. While most implementations of polariton condensation in lattices have been limited to cryogenic temperatures due to the small exciton binding energies in previous systems^26–29^, perovskite microcavities^30–32^ have recently demonstrated stable EP condensation^33^ and long-range coherent polariton condensate flow^34^ at room temperature, enabled by stable exciton formation and a high oscillator strength at room temperature. Thus, EP condensates trapped in perovskite microcavities with programmable geometric or periodic potentials show promise for the realization of topological polariton metamaterials^35^, optical Berry-phase interferometers^36^, chiral polariton lenses^37^, effective optical pendulums^38^, and polariton vortex switches^39,40^ operating at room temperature.

In this work, we have demonstrated the coupling of counterrotating polariton flows at room temperature in an artificially engineered annular potential well. In energy-momentum space, the energies of the EPs separate into multiple states with various azimuthal indices *l* and radial indices *p*, which originate from the coherent superposition of two orbital modes corresponding to clockwise and anti-clockwise rotation. Through a driven-dissipative mechanism, spontaneous petal-shaped pattern formation of the polariton density simultaneously occurs into several selected azimuthal and radial orbital states of the polariton condensates in one cylindrically symmetric ring structure. Interference measurements reveal an alternating π phase shift between neighboring lobes for these petal-shaped condensates as well as a vortex–antivortex superposition carrying a net OAM of zero localized inside the annular potential. Our results demonstrate fully collective coherent macroscopic quantum states that show promise for the realization of structured photonics and polaritonic vortices at room temperature.

## Results

**Fig. 1:**
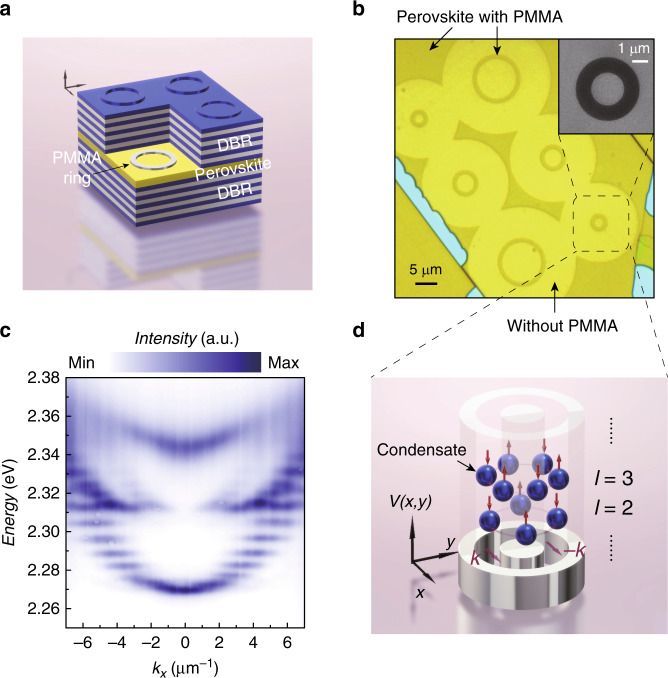
Schematic diagram and momentum-space imaging of an annular perovskite microcavity. **a** Schematic representation of an annular potential well in a CsPbBr_3_ perovskite microcavity. **b** Microscopy images of the microrings before the deposition of the top DBR, showing rings with diameters of 3, 5, and 10 μm and a width of 1 μm. Inset: scanning electron microscopy image of the 3 μm-diameter ring. **c** Experimental momentum-space polaritonic dispersion at *k*_y_ = 0 below the threshold at room temperature. The polaritonic energy is separated into multiple discrete states. **d** Schematic representation of the real-space potential profile V(*x*,*y*) and the distribution of condensates with different azimuthal indices *l*. The polariton condensate exhibits a cylindrically symmetric petal-shaped pattern and alternating phase jumps between adjacent lobes. Up and down arrows represent the *π* shifts of the condensates

Our perovskite microcavity structure is depicted in Fig. 1a. This perovskite microcavity consists of a distributed Bragg reflector (DBR) at the bottom, a gain layer of cesium lead bromide (CsPbBr_3_) perovskite, a patternable spacer layer of poly(methyl methacrylate) (PMMA) and another DBR layer on top. The PMMA spacer layer is spin coated onto the perovskite and sculpted into microrings by means of e-beam lithography to create annular potential wells to trap polaritons. As shown in Fig. 1b, there are three types of uniform microrings, with diameters of 3, 5, and 10 μm and a width of 1 μm. Figure 1c shows the momentum-space energy-resolved photoluminescence (PL) mapping of EPs imaged along the *k*_x_ direction at *k*_y_ = 0 in the annular perovskite microcavity with a diameter of 3 μm, for which the results are in agreement with the theoretically calculated dispersion (see the Supplementary Materials). The polaritonic dispersion exhibits three parts: a parabolic-like dispersion (above 2.345 eV) and upper (2.315–2.34 eV) and lower (below 2.315 eV) bands. The lower and upper bands correspond to PL emission from EPs trapped in a PMMA ring, originating from coupled orbital modes (COMs) ${|{\psi _{{\rm{p,l}}}}}\rangle$ with different azimuthal indices *l* and radial indices *p*. The parabolic-like dispersion corresponds to emission from the surrounding perovskite microcavity without PMMA, whose minimum energy is located at 2.345 eV. The potential depth can be defined as 90 meV from the energy difference between the dispersions of the surrounding planar microcavities with and without PMMA. The lower and upper bands display multiple discrete COMs, because of the elliptical effective potential originating from the anisotropic effective masses of the perovskite polaritons^22^. Every discrete COM is formed by the coupling of two pure orbital states, corresponding to opposite chiral rotations of polariton flows. The eigenstates of the COMs are defined as ${| {\psi _{{\rm{p,l}}, \pm }} } \rangle = \frac{1}{{\sqrt 2 }}( {e^{ - i\frac{{\varphi _ \pm }}{2}} {| {{p,l}_ + }} \rangle + e^{i\frac{{\varphi _ \pm }}{2}} {| {{p,l}_ - } } \rangle })$, where *φ*_±_ is the geometric phase of 0 (*π*) in anisotropic perovskites, ± corresponds to symmetric or antisymmetric combinations, and the $\left. {\left| {p,l_ \pm } \right.} \right\rangle$ are pure orbital states ($\propto e^{ \pm il\phi }$) with OAMs of $\pm l\hbar$. Only under external perturbation or symmetry breaking (i.e., a defect in the structure or an anisotropic effective mass) will the clockwise and anticlockwise modes be coupled and separated in energy. As the pumping exceeds the critical density, the EPs simultaneously condense into multiple coherent COMs via bosonic stimulated scattering, spontaneously establishing phase-ordered and phase-locked condensates, as shown in Fig. 1d.

**Fig. 2:**
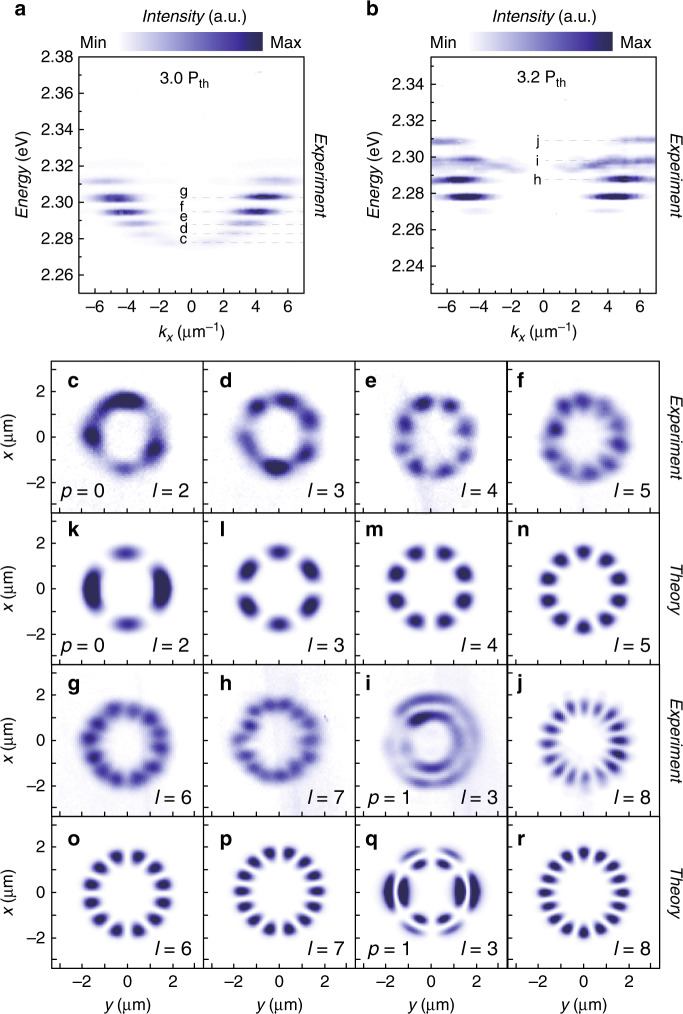
Momentum-space and real-space imaging of petal-shaped polariton condensates with vertical linear polarization at room temperature. **a**, **b** Momentum-space dispersions of polariton condensates at *k*_y_ = 0 μm^−1^ in an annular potential well at room temperature. **a**, **b** Two samples with different negative detunings under 3 *P*_th_ and 3.2 *P*_th_, respectively. The gray dashed lines represent the energy selections for the real-space imaging of the condensates. **c**–**g** Experimental real-space images of petal-shaped polariton condensates with different azimuthal indices *l*, at energies of 2.277, 2.282, 2.288, 2.295, and 2.303 eV, and a radial index of *p* = 0, corresponding to the gray dashed lines in **a**. **h**–**j** Same as **c**–**g**, at energies of 2.288, 2.298, and 2.308 eV, corresponding to the gray dashed lines in **b**. **k**–**r** Theoretical real-space images of each orbital state of the petal-shaped polariton condensates

To further elucidate the nature of the EP condensates in such an annular potential well, we recorded real-space and momentum-space images above the critical threshold (*P*_th_ ~ 12.6 μJ cm^−2^). Figure 2a, b show that under strong excitations of 3*P*_th_ and 3.2*P*_th_, respectively, the EPs condense into multiple selected COMs in two perovskite microcavity samples with different detunings (*a*: −98 meV, *b*: −130 meV), where the selected OAMs are determined by the gain-loss mechanism of non-equilibrium polariton condensation. As shown in Fig. 2c–j, the real-space images of the COMs at different energies (gray horizontal dashed lines in the symmetric dispersion images in Fig. 2a, b) exhibit stable annular petal-shaped density distributions with *n* lobes for a long time, where the number of lobes is given by *n* = 2*l*. For each given COM, two counterpropagating pure orbital states ${| {p,l_ \pm } } \rangle$ are excited simultaneously, resulting in coherently coupled standing wave patterns (due to defects, inhomogeneous pumping, or an effective mass anisotropy; see the Supplementary Materials). Under the same linear polarization, every COM possesses two mutually orthogonal patterns (they are eigenstates). As the condensate energy level rises, the number of lobes sequentially increases, and the COMs are sequentially arranged in energy from ${| {\psi _{0,2}} }\rangle$ to ${| {\psi _{0,8}}} \rangle$. When the energy of the condensates reaches a certain energy, a higher-order COM ${| {\psi _{1,3}} } \rangle$ with a radial index of *p* = 1 appears between ${| {\psi _{0,7}} } \rangle$ and ${| {\psi _{0,8}} } \rangle$, forming the double-annulus-shaped pattern seen in Fig. 2i. As shown in Fig. 2k–r, such real-space patterns can be well reproduced in theoretical calculations by solving the time-independent driven-dissipative Schrodinger equation:

1 $$\begin{array}{ll}\left( { - \frac{{\hbar ^{2} \nabla^{2} _{\rm{x}}}}{{2m_{\rm{x}}}} - \frac{{\hbar ^{2}\nabla^{2}_{\rm{y}}}}{{2m_{\rm{y}}}} + V\left( {\boldsymbol{r}} \right) + iW\left( {\boldsymbol{r}} \right)} \right) {| {\psi _{{\rm{p,l}}, \pm }} }\rangle\\ \ \ = E_{{\rm{p,l}}, \pm }{| {\psi _{{\rm{p,l}}, \pm }}}\rangle\end{array}$$

where *m*_x_ and *m*_y_ are the effective polariton masses along the two axes (the ratio of *m*_x_/*m*_y_ = 0.7 ± 0.1 accounts for the anisotropy of the perovskite)^22^, *V*(*r*) is the ring-shaped potential, and *W*(*r*) is the overall gain-loss profile, which accounts for the Gaussian-shaped non-resonant pumping and the different losses inside and outside of the ring-shaped trap. The eigenstates ${| {\psi _{{\rm{p,l}}, \pm }}}\rangle$ and eigenvalues $E_{{\rm{p,l}}, \pm }$ are obtained by diagonalizing the Hamiltonian in Eq. 1. Starting with the initial state at *t* = 0, the wavefunction ${| {\psi(t)}}\rangle$ of the whole system at any time can be decomposed into a linear combination of all eigenstates:

2 $${| {\psi(t)}}\rangle = \mathop {\sum}\limits_{{\rm{p,l}}, \pm } {C_{{\rm{p,l}}, \pm }} {| {\psi _{{\rm{p,l}}, \pm }}}\rangle e^{\frac{{ - i \cdot {\mathrm{Re}}( {E_{{\rm{p,l}}, \pm }} )t + {\mathrm{Im}}( {E_{{\rm{p,l}}, \pm }})t}}{\hbar }}$$

where the eigenvalues are separated into real and imaginary parts ($E_{{\rm{p,l}}, \pm } = {\rm{Re}} (E_{{\rm{p,l}}, \pm }) + {\rm{Im}} (E_{{\rm{p,l}}, \pm }) \cdot i$), which determine the frequency and intensity, respectively, of the field. The states with higher imaginary eigenvalues possess higher polariton populations (see the Supplementary Materials). The real-space image and reciprocal-space dispersion of the polariton condensates are represented by $| {\langle {{\boldsymbol{r}}|\psi(t)}\rangle}|^2$ and the Fourier-transformed state $| {\langle {{\boldsymbol{k}}|\psi(t)}\rangle}|^2$, respectively.

**Fig. 3:**
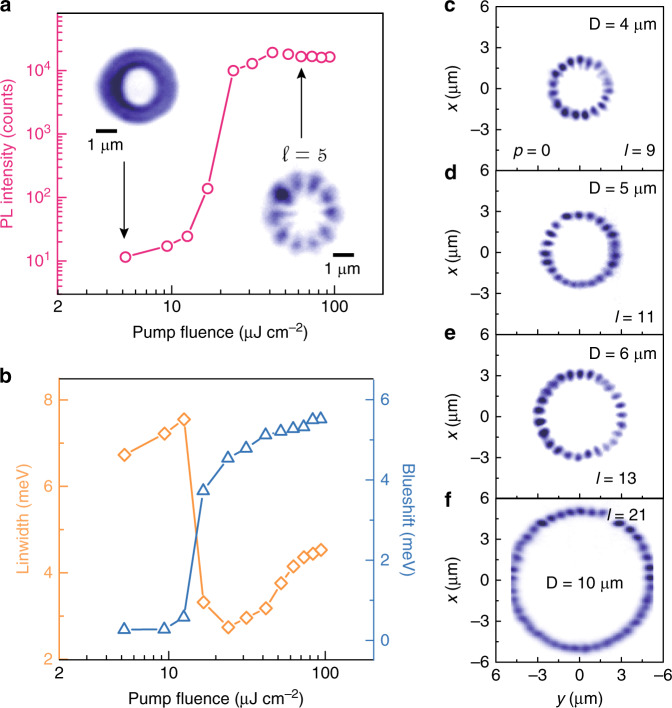
Characterization of exciton-polariton condensation. **a** Power dependence of the emitted photon flux in an annular potential with a diameter of 3 μm. Inset: real-space images of polaritons below (left) and above (right) the threshold. **b** Evolution of the emission linewidth (orange curve) and energy blueshift (blue curve) as functions of the pump fluence. **c**–**f** Experimental real-space images of petal-shaped polariton condensates in annuli with different OAMs and diameters: 4, 5, 6, and 10 μm

To quantitatively characterize the formation process of the EP condensates, we demonstrate the evolution of the PL intensity, linewidth, and energy blueshift, which are extracted from the emission spectra of the condensation state at the fixed in-plane wavevector as functions of the pump fluence. In Fig. 3a, when the pump fluence crosses the threshold of *P*_th_ = 12.6 μJ cm^−2^, an unambiguous superlinear increase in the PL intensity by three orders of magnitude is observed, enhanced by the stimulated scattering of polaritons directly from the pumping position. In Fig. 3b, the polariton emission first exhibits an increase in linewidth to 7.5 meV below the threshold and then evolves abruptly into a narrow peak with a linewidth of 2.7 meV at the threshold, indicating a spontaneous build-up of temporal coherence in the polariton condensates. The non-resonant pumping excites the global exciton reservoir; subsequently, the excitons relax and couple to geometrically constrained photon modes, enabling the trapping of polaritons in the annulus. In the low-polariton-density regime, the polariton emission has a doughnut shape (left inset of Fig. 3a) due to an incoherent superposition of many weakly populated modes in the entire annulus below the threshold, with an intensity of $\mathop {\sum}\nolimits_{{\rm{p,l}}, \pm } {\left| {\left. {\left| {\psi _{{\rm{p,l}}, \pm }} \right.} \right\rangle } \right|} ^2{\mathrm{exp}}\left( {\frac{{ - E_{{\rm{p,l}}, \pm }}}{{k_{\rm{b}}T}}} \right)$. In a sufficiently high-density regime, the polariton population reaches and exceeds a critical condensation density, and polariton condensates spontaneously form coherent petal-shaped patterns in multiple selected COMs. With increasing pump fluence, the polariton emission energy exhibits a continuous blueshift due to the nonlinear repulsive polariton–polariton and polariton–exciton reservoir interactions in the annular potential well. Modulating the diameter of the annulus by patterning during fabrication enables the formation of petal-shaped condensates with an arbitrary even number of lobes, as shown in Fig. 3c–f. The geometric parameters of the annular potential determine the energy interval between adjacent discrete COMs. A larger ring diameter gives rise to a smaller energy interval and a larger azimuthal or radial index of the observed condensates (see the Supplementary Materials). The lateral cut-off boundary in the image in Fig. 3f is attributed to the signal exceeding the collection range of the spectrometer slit.

**Fig. 4:**
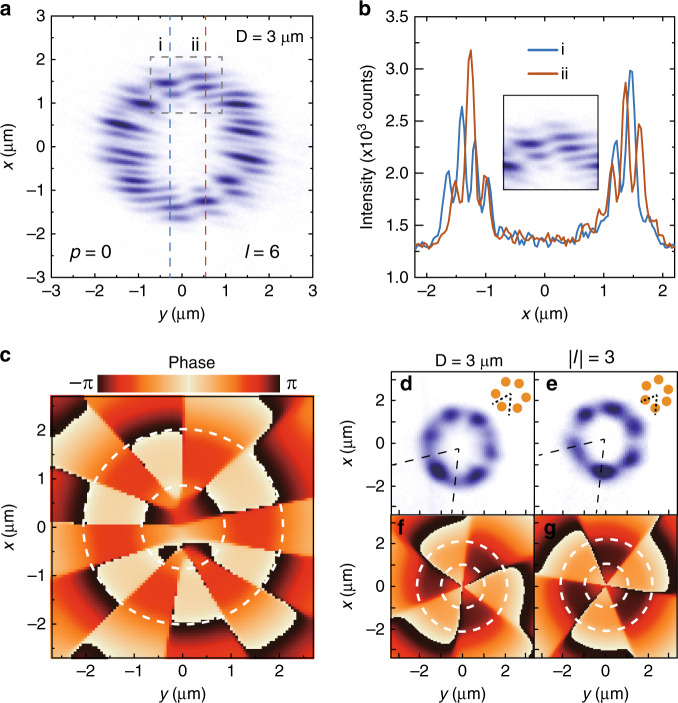
Interference and phase of the petal-shaped polariton condensates. **a** Interferogram of a petal-shaped polariton condensate above the threshold in an annular potential well. **b** Interference spectra extracted from **a**, corresponding to the orange and blue dashed lines, confirming the π phase shift between adjacent lobes. Inset: zoomed image of the dashed box in **a**. **c** Theoretically calculated phase of the condensate corresponding to **a**; the white dashed concentric circles represent the profile of the annulus. **d**, **e** Experimental real-space images of two petal-shaped polariton condensates with mutually orthogonal linear polarization. **f**, **g** Theoretically calculated phases corresponding to **d** and **e**. **d**, **e** represent the COMs ${| {\psi _{0,3, + }}}\rangle _{\rm{V}}$ and ${| {\psi _{0,3, - }}}\rangle _{\rm{H}}$, respectively, in which the patterns are orthogonal to each other. The orange balls in the insets show the geometric symmetry of the distribution of the condensate lobes

To study the phase distribution of the wavefunction of the petal-shaped condensates in such an annular perovskite microcavity, we characterized the interferogram of the COMs with a radial index of zero. EP condensation is accompanied by a phase transition from thermal phases below the threshold to a condensed phase above the threshold, which indicates a spontaneous build-up of long-range order manifesting as collective coherence covering the entire condensate region. In our experiment, the real-space image of the petal-shaped condensates above the threshold was sent into a Michelson interferometer, and the signal superimposed with its centrosymmetrically inverted image was collected. Figure 4a exhibits clear interference fringes of ${| {\psi _{0,6}}}\rangle$ throughout the whole annulus region, manifesting the spontaneous build-up of long-range spatial coherence between two centrosymmetric points on the ring. The interference fringes between adjacent lobes of the petal-shaped polariton condensates are discontinuous and staggered into stripes (Fig. 4b), which implies the existence of a *π* phase shift between each pair of adjacent lobes. Since *l* = 6 is an even number, the phases between the original and centrosymmetric inverted lobes are consistent. Due to the minor displacement between two centrosymmetric images (see the Supplementary Materials), simultaneous overlapping of one lobe with its two neighboring lobes with a *π* phase difference results in the staggered interference fringes of the polariton condensates. In Fig. 4c, the corresponding phase simulation of the condensates shows that each lobe of the 12-lobe COM possesses a well-defined phase and theoretically exhibits a phase shift on the order of *π* between every two adjacent lobes (as shown by the phase inside the annulus, outlined by white dashed concentric circles), stemming from the periodic boundary conditions constraining the wavefunctions. Here, the polariton density of the interference image is defined as:

3 $$I = \left| {\left\langle {\left. {\boldsymbol{r}} \right|\psi _{{\rm{p,l}}}} \right\rangle + \left\langle - {\left. {\boldsymbol{r}} \right|\psi _{{\rm{p,l}}}} \right\rangle e^{i{\boldsymbol{k}} \cdot {\boldsymbol{R}}}} \right|^2$$

where $\langle - {\boldsymbol{r}}|\psi _{{\rm{p,l}}}\rangle$ is the centrosymmetric inverted state and ***R*** is the optical path difference between the original and inverted images. Moreover, every single pure orbital mode possesses an OAM with a helically propagating phase promising a vortex core^7^; thus, ${| {p,l_ + }}\rangle$ and ${| {p,l_ -}}\rangle$ carry left-handed and right-handed vortices, respectively, with opposite topological charges of ±l. However, the net OAMs of their coupled states ${| {\psi _{\rm{p,l}}}}\rangle$ are zero, leading to a vortex–antivortex superposition in the center of the annulus, which proves that the petal-shaped condensates originate from the coherent coupling of two opposite rotational states. At low momenta, the linear polarization splitting is dominant over the effective mass anisotropy. Consequently, the COMs possess different linear polarizations for a given OAM $\left| l \right|$; two spatially orthogonal patterns can arise for each given polarization (due to disorder or the mass anisotropy itself). When linear polarization splitting is considered in the system, Eq. (1) is modified as follows:

4 $$\begin{array}{ll}\left( { - \frac{{\hbar ^{2}\nabla^{2}_{\rm{x}}}}{{2m_{\rm{x}}}} - \frac{{\hbar ^{2}\nabla^{2}_{\rm{y}}}}{{2m_{\rm{y}}}} + V\left( {\boldsymbol{r}} \right) + iW\left( {\boldsymbol{r}} \right)} \right)\left. {\left| {\psi _{{\rm{p,l}}, \pm }} \right.} \right\rangle _{{\rm{V,H}}} \\ \,\,\,\, +\, \delta \left. {\left| {\psi _{{\rm{p,l}}, \pm }} \right.} \right\rangle _{{\rm{H,V}}} = E_{{\rm{p,l}}, \pm }\left. {\left| {\psi _{{\rm{p,l}}, \pm }} \right.} \right\rangle _{{\rm{V,H}}}\end{array}$$

where *δ* is the strength of the linear polarization splitting and *V* and *H* represent the vertical and horizontal linear polarizations, respectively; more details are shown in the Supplementary Materials (Fig. S8). Figure 4d, e exhibit two orthogonal linearly polarized real-space patterns of the same OAM ($\left| l \right| = 3$), which are ${| {\psi _{0,3, + }}}\rangle _{\rm{V}}$ and $| {{\psi _{0,3, - }}\rangle }_{\rm{H}}$ and display orthogonal pattern distributions. These two COM eigenstates can be represented by two endpoints of the *x*-axis on the equator of the OAM Poincaré sphere for linear polarization^9,11^. By utilizing polarization splitting and symmetry breaking, one can manipulate the eigenstates to create more orbital states on the OAM Poincaré sphere. Such spontaneously formed patterns of polariton condensates unambiguously demonstrate a symmetric and alternating antiphase ordering.

## Discussion

In summary, we have implemented the coupling of counterrotating EP flows in an artificial engineered annular perovskite microcavity at room temperature. The coupling of two opposite rotational orbital modes leads to the formation of a phase-locked petal-shaped pattern in real space and multiple discrete EP condensate states in momentum space, due to the symmetry breaking caused by the anisotropic effective mass. These patterns of coupled states display stable alternating *π* phase shifts between neighboring lobes as well as a vortex–antivortex superposition localized in the core of the annulus, which is highly susceptible to defects and potential disorder. The investigation of such macroscopic quantum states with collective coherence in a solid-state system can facilitate the study of structured photonics and polaritonic vortices at room temperature. The scheme used to pattern the polariton potentials into arbitrary geometries lays the foundation for exploring optical topological polariton devices and polariton switches operating at room temperature.

## Materials and methods

### Perovskite microcavity fabrication

The bottom DBR, consisting of 30.5 pairs of TiO_2_ and SiO_2_, was grown using an electron beam evaporator. The perovskite layer was transferred onto the bottom DBR via a dry-transfer method using cellophane tape. The growth of CsPbBr_3_ perovskite single crystals has been described in our previous reports^33^. Then, a 61 nm-thick PMMA spacer layer was spin coated onto the perovskite layer and patterned via electron beam lithography. Finally, the top DBR, consisting of 10.5 pairs of Ta_2_O_5_ and SiO_2_, was fabricated with the electron beam evaporator.

### Optical spectroscopy characterization

The real-space and momentum-space PL images of polariton emission were detected using a micro-PL setup with a Fourier imaging configuration, collected through a 50× objective, and then sent to a spectrometer equipped with a grating of 600 lines/mm and a liquid nitrogen-cooled charge-coupled device of 256 × 1024 pixels. In the linear regime, the perovskite microcavity was non-resonantly excited by a continuous wave laser of 457 nm with a pump spot of ~15 μm. In the nonlinear regime, real-space and momentum-space images of the samples were characterized under non-resonant excitation using a pulsed laser of 400 nm, with a pump spot of ~21 μm. The pulse duration was 100 fs, and the repetition rate was 1 kHz. Energy-selected real-space images were measured using a narrow laser line filter with a linewidth of approximately 1 nm on the detection path. The long-range spatial coherence was measured using a Michelson interferometer (the details of the setup are shown in the Supplementary Materials).

## Supplementary information

Supplementary Information for Spontaneously Coherent Orbital Coupling of Counterrotating Exciton Polaritons in Annular Perovskite Microcavities
