# Supplementary material for: Spontaneously coherent orbital coupling of counterrotating exciton polaritons in annular perovskite microcavities
Source: Light Sci Appl. 2021 Mar 1;10:45. doi: 10.1038/s41377-021-00478-w (PMC7921445; doi:10.1038/s41377-021-00478-w)
Supplement: Supplementary file 1 — Supplementary Information for Spontaneously Coherent Orbital Coupling of Counterrotating Exciton Polaritons in Annular Perovskite Microcavities [file 41377_2021_478_MOESM1_ESM.pdf]

Supplementary Information for

# Spontaneously Coherent Orbital Coupling of Counterrotating Exciton Polaritons in Annular Perovskite Microcavities

Jun Wang,[1,⊥] Huawen Xu,[1,⊥] Rui Su,[1,*] Yutian Peng,[2] Jinqi Wu,[1] Timothy C. H. Liew,[1,3,*] and Qihua Xiong[2,4,*]

[1]Division of Physics and Applied Physics, School of Physical and Mathematical Sciences, Nanyang Technological University, 637371, Singapore.

[2]State Key Laboratory of Low-Dimensional Quantum Physics and Department of Physics, Tsinghua University, Beijing, P.R. China.

[3]MajuLab, International Joint Research Unit UMI 3654, CNRS, Université Côte d'Azur, Sorbonne Université, National University of Singapore, Nanyang Technological University, Singapore, Singapore.

[4]Beijing Academy of Quantum Information Sciences, Beijing 100193, P.R. China

⊥These authors contributed equally: Jun Wang, Huawen Xu.

*To whom correspondence should be addressed. E-mail: Qihua_xiong@tsinghua.edu.cn; Surui@ntu.edu.sg; and Timothyliew@ntu.edu.sg

## 1. Dispersion simulation and fluorescence image

Fig. S1a represents the theoretical momentum-space polaritonic dispersion of the ring with a diameter of 3 μm, in good agreement with the experimental data of Fig. 1c of the text. The polaritonic energy separates into multiple discrete coupled orbital modes (COMs). This is obtained from a Fourier transform of the wavefunction $\psi(t,x,y)$ into the energy domain in reciprocal space $\psi(E,k_x,k_y)$. Theoretically, the reciprocal-space dispersion is given by $I = |\psi(E,k_x,k_y=0)|^2$, which is a function of $k_x$ and $E$ at $k_y = 0$.

The fluorescence image of the micro-rings was obtained through an Olympus microscope, where the sample was illuminated by an Olympus U-HGLGPS lamp. In Fig. S1b, bright green areas are covered by PMMA without exposure, dark green areas are not covered by PMMA with exposure. Due to the quenching of the sample fluorescence by electron beam lithography, the photon emission from unexposed areas with PMMA is brighter than the exposed areas without PMMA. Thus, one can speculate that the Q-factor inside the ring structure is higher than that of the outside. Consequently, we take into account a different local lifetime inside and outside the potential well in our theoretical model.

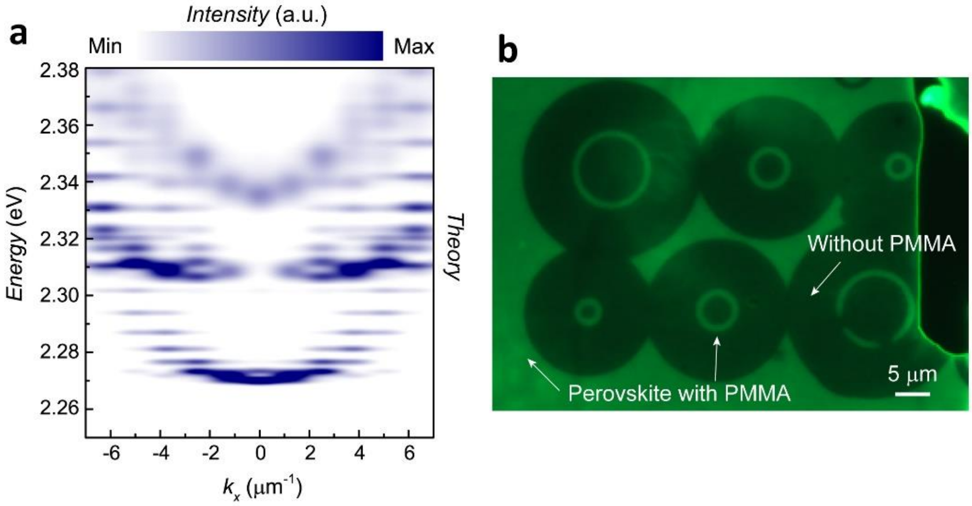


**FIG. S1** (a) Theoretical momentum-space polaritonic dispersion of 3 μm ring at $k_y = 0$ below the threshold at room temperature. (b) Microscopy fluorescence images of the micro-rings before the deposition of top DBRs, showing the rings with diameters of 3 μm, 5 μm, 10 μm, and width of 1 μm.

## 2. Comparison of dispersion

The geometric parameters of the annular potential determine the energy intervals of adjacent discrete COMs. Comparing Fig. S2a and c, we can observe the energy intervals of adjacent discrete COMs in both the lower band of $p = 0$ and the middle band of $p = 1$ are different, where p is the radial quantum number. The larger the diameter of the ring, the smaller the energy interval, and the greater the number of adjacent discrete COMs. The theoretical simulations are consistent with the experimental data, evidencing this phenomenon.

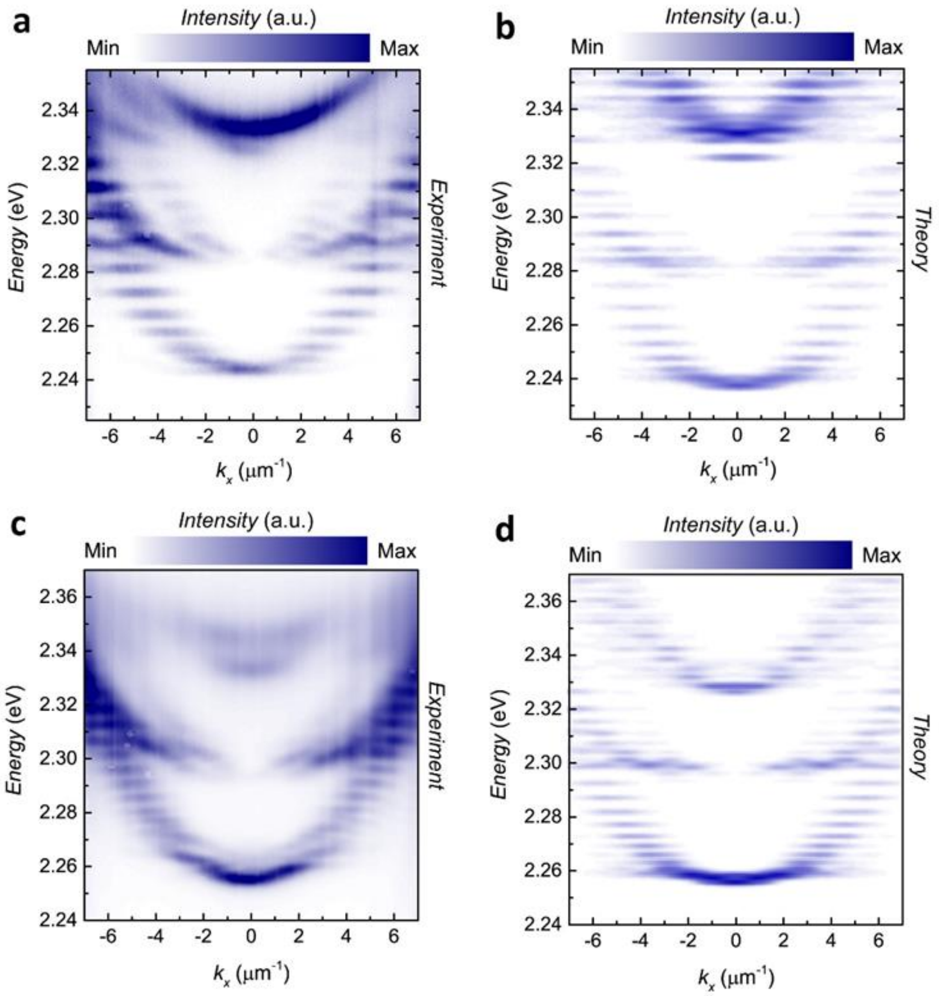


**FIG. S2** (a, c) Experimental momentum-space polaritonic dispersion at $k_y = 0$ below the threshold at room temperature, in the micro ring with a diameter of 3 μm for (a) and 5 μm for (b), respectively. (b, d) Theoretical momentum-space polaritonic dispersion, corresponding to (a) and (c), respectively.

## 3. Gain-loss mechanism

Combining Eq. 1 and Eq. 2 of the text, we can expect that the real part of eigenvalue determines the frequency of the field, and the imaginary part of eigenvalue determines the gain. The state with a higher imaginary eigenvalue (higher gain) will have a higher polariton population. As shown in Fig. S3, we show how the imaginary part of the eigenvalue evolves with the real part of the eigenvalue. The states with real parts of eigenvalues from 2.95 eV to 3.05 eV possess higher imaginary eigenvalues than other states, which implies massive polariton condensates will occupy in such states. In Fig. S3, the states above the dashed line possess higher gain, which indicates where the condensation occurs.

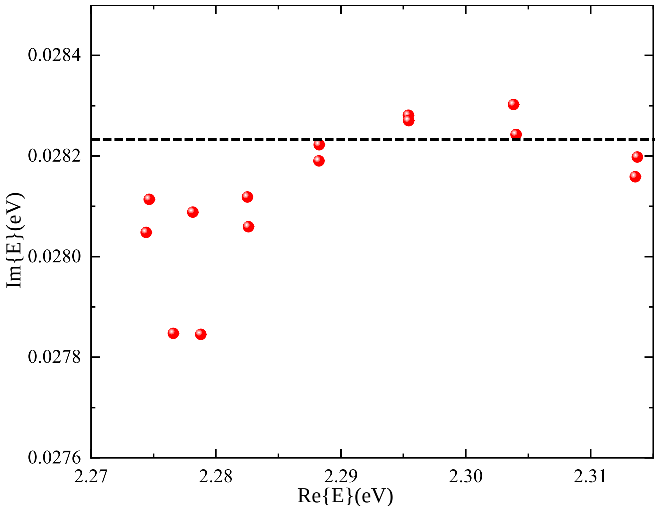


**FIG. S3** The relationship between the imaginary part of eigenvalue (Im$\{E\}$) and the real part of eigenvalues (Re$\{E\}$).

# 4. Polariton condensates of the 5 μm ring

As the mode energy increases, for the COMs $|\psi_{0,6}\rangle$ to $|\psi_{0,11}\rangle$, the number of lobes sequentially increases. When the energy of condensates reaches certain energy, a superposition state of higher-order COMs with a radial index of $p = 1$ appears, forming a double-annulus-shaped pattern of Fig. S4h. It is worth mentioning that the patterns of $|\psi_{0,6}\rangle$ and $|\psi_{0,7}\rangle$ modes are a little asymmetric, and some lobes are displaced. The reason is the external perturbation or potential symmetry breaking (*i.e.*, a defect or a mutation in structure), resulting in the two energy degenerate modes slightly separating in energy. Nevertheless, the coupling of condensate flows can still stably form the standing-wave pattern throughout the entire annulus, and the pattern symmetry is affected by the defect, which is highly sensitive to defects and potential disorder.

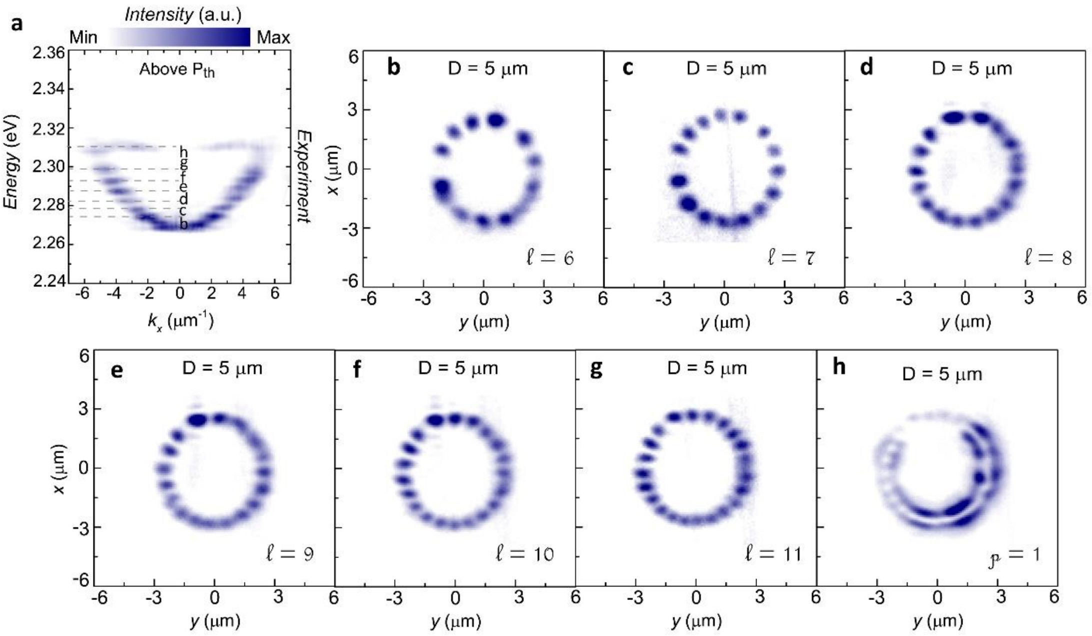


**FIG. S4** (a) Momentum-space dispersions of polariton condensates of a 5 μm micro ring at $k_y = 0$ in annular potential well above the threshold at room temperature. The gray dashed lines represent the energy selections of the real-space imaging of condensates. (b-h) Experimental real-space images of petal-shaped polariton condensates with different azimuthal index $l$ (from 6 to11) and radial index $p = 1$ in the micro ring with a diameter of 5 μm, corresponding to the gray dashed lines in (a).

## 5. Polariton condensates of the 10 μm ring

The geometric parameters of the annular potential determine the number of lobes. The larger the diameter of the ring, the more the number of lobes, and the larger the azimuthal and radial index of observed condensates, as shown in Fig. S5. The observed petal-states with a single ring are originated from the coherent coupling of two pure orbital modes with zero radial and opposite azimuthal indices, and the relationship between the number of lobes and azimuthal index is $n = 2l$.

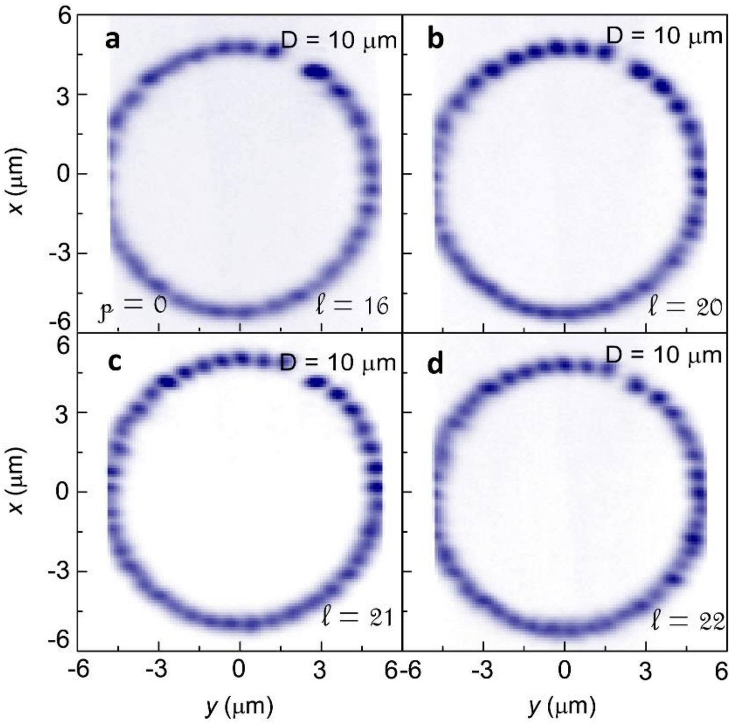


**FIG. S5** (a-d) Experimental real-space images of petal-shaped polariton condensates with different azimuthal index $l$ ($l$=16, 20, 21, and 22) and radial index $p$ = 0 in the micro ring with a diameter of 10 μm.

## 6. Degenerate antisymmetric pattern

Each energy level of condensate states possesses two orthogonally polarized and energetically degenerate antisymmetric patterns. Two counterpropagating exciton polariton flows are excited simultaneously, resulting in the observed standing wave patterns. The geometry in Fig. S6b is orthogonal with Fig. S6a. The phase distributions of both Fig. S6c and d display a stable alternating π phase jump between neighboring lobes, as well as a vortex-antivortex superposition localized in the core of the annulus.

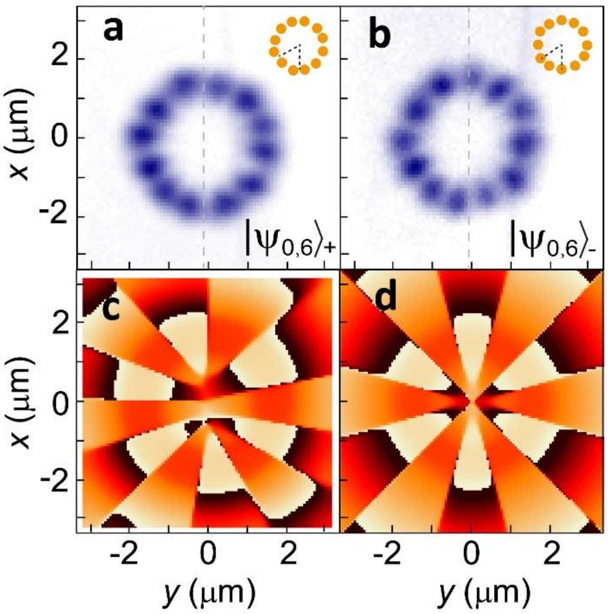


**FIG. S6** (a, b) Experimental real-space images and (c, d) the phase of two-fold degenerate petal-shaped polariton condensates in the annulus with a diameter of 3 μm. The azimuthal index is six ($p$ = 0, $l$ = 6). The insets are the distribution of geometric symmetry for the lobes of the petal-shaped condensates. The orange balls in the insets of (a) and (b) represent the lobes.

## 7. The Setup of Michelson interferometer

The emergence of long-range spatial coherence of polariton condensates can be demonstrated by a Michelson interferometer with a retroreflector in one arm, which allows a centrosymmetric inverting image, as shown in Fig. S7a. In our sample, the real-space image of petal-shaped condensates ($p = 0$, $l = 6$) above the threshold is sent into the interferometer, then the signal superimposed with its reverted image is collected. The interference fringe contrast is the manifestation of phase coherence between points **r** and **-r** with respect to the center. In the simulated interferogram image of Fig. S7b, there is a clear shift of the interference fringes between adjacent lobes, which agrees with the experimental result of Fig. S7e. When there is a minor displacement (as shown in the dashed area of Fig. S7c and d) between the polariton condensate image and its inverted image after superposition, simultaneously overlapping between one lobe and the other two adjacent lobs could lead to clear interference patterns with a π phase shift in the middle of two adjacent lobes. This minor displacement is due to the uneven distribution of lobes for polariton condensates in the annular potential.

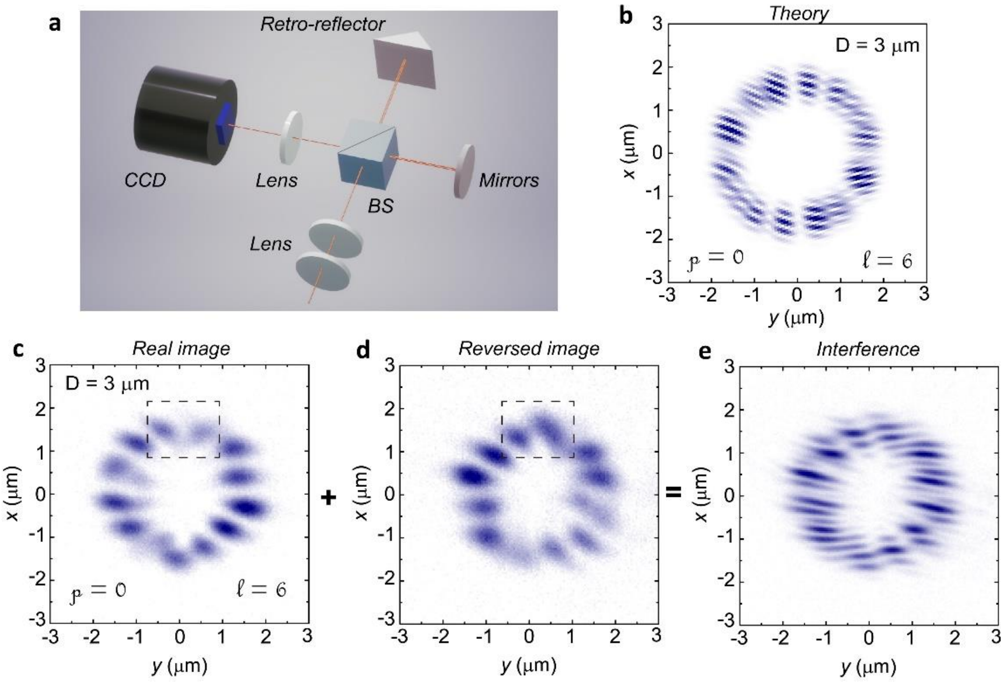


**FIG. S7** (a) The schematic of the experimental Michelson interferometer setup. (b) The simulated interferogram, (c) the real space image, (d) its inverted image, and (e) the interferogram of the petal-shaped polariton condensate ($p = 0$, $l = 6$) above threshold in the annular potential well.

## 8. Orbital angular momentum Poincaré sphere representation of the degenerate states

The arbitrary state $|v\rangle$ in orbital angular momentum (OAM) Poincaré sphere (PS) representation can be described by left-rotation eigenvector $|l_+\rangle$ and right-rotation eigenvector $|l_-\rangle$, as following:

$$|v\rangle = e^{-i\frac{\varphi}{2}}\cos\frac{\theta}{2}|l_+\rangle + e^{i\frac{\varphi}{2}}\sin\frac{\theta}{2}|l_-\rangle$$

where $\theta$ and $\varphi$ are the polar coordinates of OAM PS, the states of OAM PS can be represented by a vector $(\theta, \varphi)$. Two degenerate coherent coupling states ($|\psi_+\rangle$ and $|\psi_-\rangle$) of the integer orbital angular momentum modes in our perovskite microcavity can also be unfolded by eigenvectors $|l_+\rangle$ and $|l_-\rangle$ of OAM PS representation in the linear polarization. For example, $|\psi_{0,3}\rangle_+ = \frac{1}{\sqrt{2}}(|0,3\rangle + |0,-3\rangle)$ and $|\psi_{0,3}\rangle_- = \frac{-i}{\sqrt{2}}(|0,3\rangle - |0,-3\rangle)$ are represented on two points of OAM PS (π/2, 0) and (π/2, π). Thus, two eigenvectors both $|\psi_+\rangle$ and $|\psi_-\rangle$ can be represented on two endpoints of the x-axis of the equator of OAM PS, and they are orthogonal with each other, as shown in Fig. S8. If the perturbation or symmetry broken is introduced into this system, one can create more orbital states on PS.

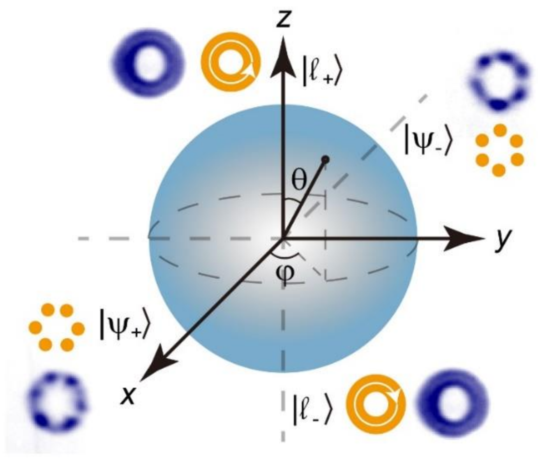


**FIG. S8** The schematic of pure orbital states $|l_\pm\rangle$ and COMs $|\psi_\pm\rangle$ in OAM Poincaré sphere representation.

## 9. The influence of linear polarization for petal-like polaritons

In the annular microcavity, we have measured the angle-resolved PLs with the different polarizations (Fig. S9a and b), which show the energies of horizontal (H) polarization modes are higher than the corresponding vertical (V) polarization modes, due to the V-H linear polarization splitting (~10 meV at zero-momentum). In the theoretical calculation, we take the linear polarization splitting from the birefringence effect into consideration, then introduce the linear polarization splitting strength $\delta$ into Eq. 1 of the main text as follows:

$$E\varphi_{\pm,p,l} = \left(-\frac{\hbar^2\nabla_x^2}{2m_x} - \frac{\hbar^2\nabla_y^2}{2m_y} + V(r) + iW(r)\right)\varphi_{\pm,p,l} + \delta\varphi_{\mp,p,l}$$

where $\pm$ indicates the linear polarization direction. By diagonalizing the revised Hamiltonian of the system, two sets of eigenstates in different polarization direction (plus and minus) are obtained, where the energy splitting between two orthogonal linear polarizations is around 10 meV as shown in Fig. S9c, in agreements with the dispersions in the annular and planar cavity. The two linear polarizations can be split in energy and then considered as separate. Moreover, under the same linear polarization, each orbital mode has two eigenstates which are corresponding to two antisymmetric-distribution petal-like patterns, their energies have a splitting (smaller than the linewidth) which originates from the anisotropic effective mass in birefringence perovskite. The anisotropic effective mass distorts the circular spatial potential into an elliptical effective potential, creating a symmetry breaking and resulting in the coupling. Compared with the isotropic case (Fig. S9d, we can see the small energy splitting of two eigenstates disappears. The emission probabilities of the eigenvalues are different because the random noise of the pumping spot and microcavity reflectivity affects the lifetime of every eigenstate. Therefore, sometimes two patterns with antisymmetric distributions will have different linear polarizations.

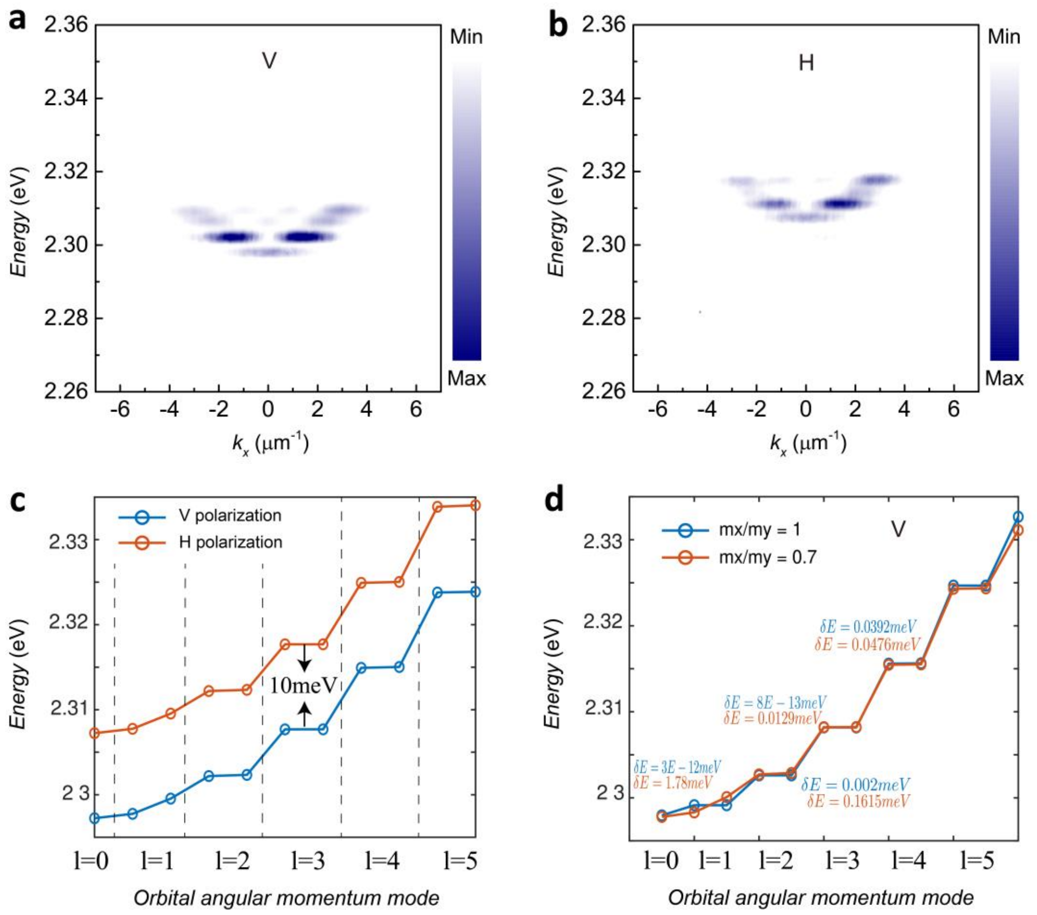


**FIG S9.** Momentum-space dispersions of polariton condensates at $k_y = 0\ \mu\mathrm{m}^{-1}$ in an annular potential well at room temperature. **a**, Vertical linear polarization modes. **b**, Horizontal linear polarization modes. **c**, Energy versus modes in the theoretical calculation, blue: vertical polarization, red: horizontal polarization. In every OAM, there are two antisymmetric-distribution patterns. **d**, Energy versus modes in the theoretical calculation of isotropic and anisotropic system.

## 10. The transition from the strong to the weak coupling regime

To prove the transition from a strong coupling region to a weak coupling region on the annular sample, we have measured the intensity, linewidth, and spatial image of polaritons emission from low pump fluence to ultrahigh pump fluence. Our system is more likely to reach a sufficiently high carrier density in the reservoir for the formation of an electron-hole plasma (EHP) and the appearance of an eventual photonic lasing process in the weak coupling regime.

From the evolution of dispersions, at $P$ = 35 $P_{th}$, Fig.S10b shows some weak light emissions leaked from the additional uncoupled cavity at lower energy than the polariton branch, which are originated from the reflections between two edges of the perovskite nanoplatelet. When further increasing the pump fluence (at $P$ = 56 $P_{th}$), the emissions from the uncoupled cavity of edges are characterized by a flat dispersion, while the emission of the lower polariton branch of the annular cavity is still visible with a much lower intensity. In real space, with increasing the pump fluence (at $P$ =35 $P_{th}$), the petal-like condensate is still visible, but the weak emission leaked from edges can be observed at the bottom of Fig. S10e. When the pump fluence approaching the ultrahigh value, the petal-like condensate emission presents much low intensity even disappeared, on the contrary, the emission leaked from the uncoupled cavity of edges is dominating. We attribute such additional emission to the EHP emission in the weak coupling regime, which is accompanied by a band-gap renormalization at high pump fluence as previously observed in ZnO microcavities for example (*New J. Phys.* **14**, 013037 (2012)). The band-gap renormalization shifts the gain of perovskites to lower energy while the cavity mode from the vertical-cavity is far above that cannot couple to the gain of perovskite in the weak coupling regime. In this case, the horizontal cavity provided by the two end facets of the perovskite nanoplatelet can provide such cavity modes to couple the perovskite gain in the weak coupling regime as a consequence of long cavity length (lateral dimension of perovskite nanoplatelet). This can be further confirmed from the real space emission images. Apart from the flat dispersion of the EHP emission, we also observe a very different emission diagram in the real space since the emission from the edge of the belt-type perovskite platelet dominates at ultrahigh pump fluence (at $P$ = 56 $P_{th}$). With these results, the polariton emission in the strong coupling regime can be clearly distinguished from the EHP emission in the weak coupling regime.

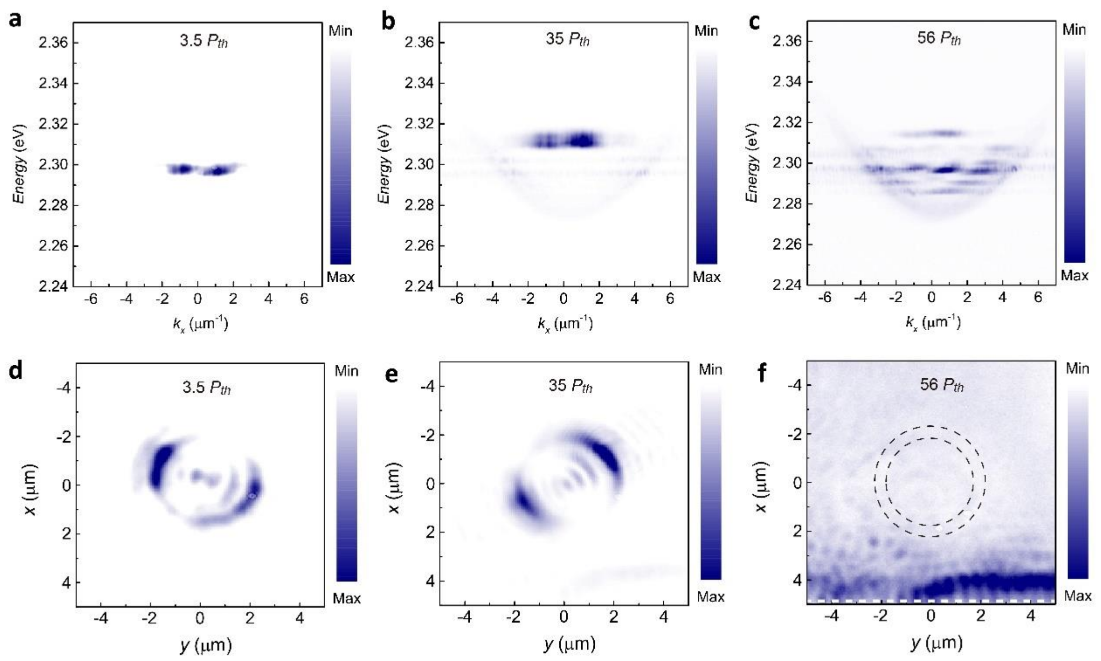


**FIG S10. Transition from strong coupling to weak coupling regime. a-c**, Angle-resolved PL spectrum obtained at 3.5 $P_{th}$, 35 $P_{th}$ and 56 $P_{th}$, showing a transition from polariton condensate regime to weak coupling regime with a sharp decrease of emission intensity. **d-f**, Real-space images obtained at 3.5 $P_{th}$, 35 $P_{th}$ and 56 $P_{th}$, showing the two-lobes polariton condensate with OAM of 1. Further increasing the pump fluence to 56 $P_{th}$, the polariton condensate vanishes while an extremely stronger emission appears at the edges of the perovskite platelet in the microcavity.

To better understand the emission properties observed in the strong and weak coupling regimes, we have analyzed the power dependence of the emission intensities, linewidths, and the emission intensities of the edge in Fig. S11. We first observe a transition associated with a sharp increase of the polariton emission intensity, and a narrowing of the emission linewidth in the strong coupling regime. Then, the increase of the pump fluence further far exceeds the polariton lasing threshold, i.e., above $P_{th2}$ =310 μJ cm$^{-2}$, leading to the polariton condensate vanishing and the related decrease of the emission intensity at the energy. This decrease in emission intensity could be explained by the change of cavity direction from vertical to horizontal. Meanwhile, the EHP emission appears and dominates the signal above $P_{th2}$, mainly at the perovskite edges. Fig. S11c defines a second threshold for the transition to the weak coupling regime in the edge emissions of the sample. This statement is further confirmed by the superlinear behavior of the EHP emission intensity above $P_{th2}$.

With these additional results, we have brought clear proof that the nonlinear behavior observed in our perovskite samples corresponds to a polariton lasing effect induced by the formation of a polariton condensate, and not to the more classical photonic lasing effect observed in the weak exciton-photon coupling regime. The transition from a strong coupling region (polaritonic lasing) to a weak coupling region (photonic lasing) is supported by the dispersions, spatial images, and the power dependence measurements.

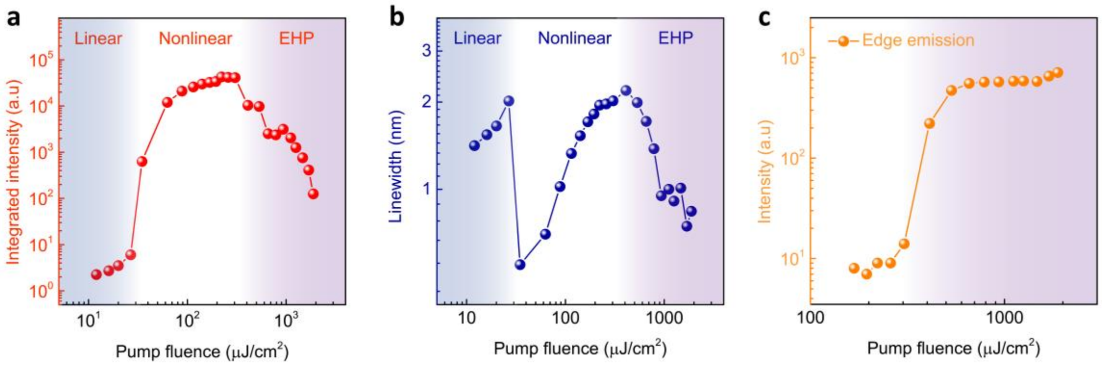


**FIG S11. Characterizations of polariton lasing in the annular perovskite microcavity. a**, **b**, the emission intensity and linewidth of the state with OAM of 1, at $k$ = 0 as a function of pump fluence. A typical S shape power-dependent emission relationship suggests the occurrence of polariton lasing with a first threshold $P_{th}$ of 26.7 μJ cm$^{-2}$, while the emission intensity decreases sharply crossing a pump fluence $P_{th2}$ of 310 μJ cm$^{-2}$. The linewidth first slightly increases below threshold $P_{th}$ and narrows from 2.017 nm to 0.55 nm when crossing the threshold $P_{th}$, and then decreases with the further increase of pump fluence. **c**, Edge emission intensity as a function of pump fluence, extracted from the real space images of the perovskite microcavity. A clear second threshold $P_{th2}$ of 310 μJ cm$^{-2}$ was observed for the emission in the lateral cavity modes.

## 11. Linear polarization splitting in a birefringent perovskite microcavity

At room temperature, our perovskite is known to be orthorhombic with birefringent effects, which has been shown to support anisotropic polaritons before (*Nat. Phys*. **16**, 301 (2020)). Here, we provide more experimental evidence regarding the birefringence effect of this system. We can define the long (Y) and short (X) axes from the optical microscopy image of Fig. S12a. The birefringent behavior in our system could also be recognized from the polarization-resolved polariton dispersions. In the planar microcavity, Fig. S12b shows the zero wavevector emission has energy splitting up to 11 meV under V and H linear polarization, which arises from the birefringent effect of the $CsPbBr_3$ perovskite. The ratio between the two effective masses is around 0.7 from the fitting, which is in good agreement with the previous report. The same energy splitting could also be observed for the crystal X axis under the V and H polarizations. The curvatures of these four dispersions ($Y_V$, $Y_H$, $X_V$, $X_H$) are different, indicating the relationship of four effective masses is $X_V < Y_V < Y_H < X_H$. There are two kinds of splitting in our system. One is the linear polarization splitting which enables V and H linear polarization mode to split in energy. The other one is the polariton dispersion with an elliptic crossed section, *i.e.*, anisotropic effective mass, which is significant to the counterpropagating polariton coupling and their spatial pattern distribution.

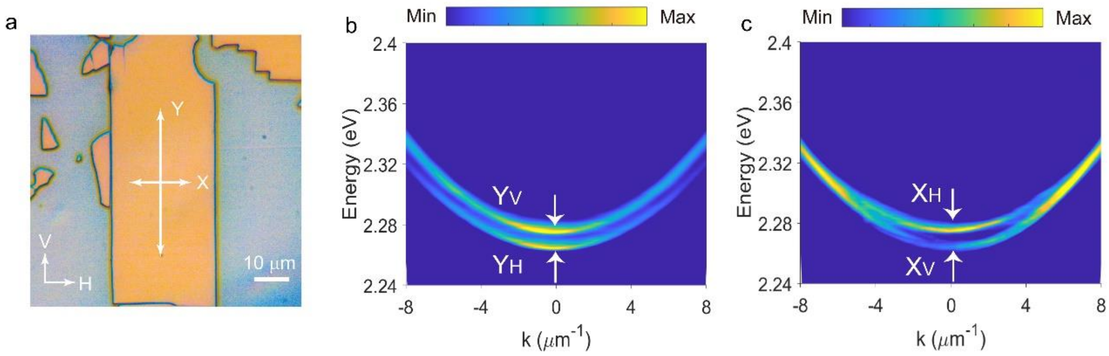


**FIG S12. a**, A typical perovskite planar microcavity with a clear crystal axis. The long (Y) and short (X) axes of the crystal. Polarization parallel (V) and perpendicular (H) to the Y-axis. **b**, Polariton dispersions along the Y-axis were obtained under V and H polarizations. The extracted parameters are $E_{YV}(0) = 2.275$ eV, $E_{YH}(0) = 2.264$ eV and $m_{YV}/m_{YH} = 0.7$. **c**, Polariton dispersions along the X-axis were obtained under V and H polarizations.

## 12. The propagation of polaritons in the 10 μm annular microcavity

To prove the propagation of polaritons, we have focused the femtosecond pulse laser with the smaller spot (1 μm) on the left side of the annulus (diameter: 10 μm) to excite the sample (Fig. S13). An unambiguous long-range propagation and a clear petal-like pattern are demonstrated by the spatial image. The coupled modes for a given OAM are populated, polaritons will have propagation around the ring. The petal-like pattern does not depend on the size and position of the excitation spot, the long-range propagation is supported by the experiments of the excitation covering one side of the ring and the whole ring.

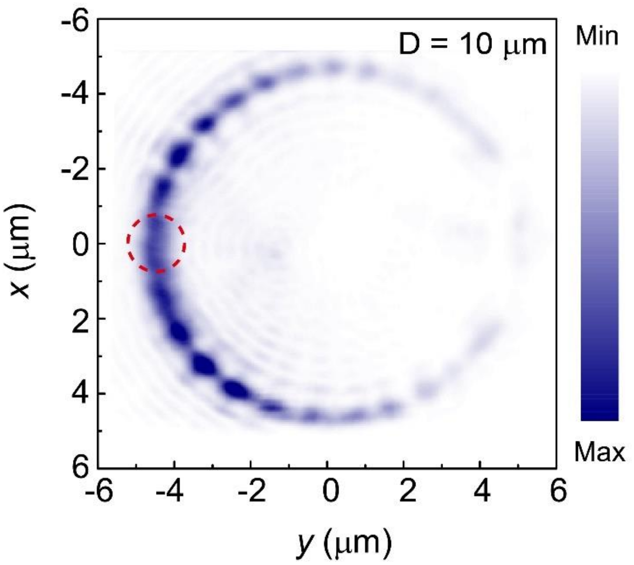


**FIG S13.** Real-space image of the petal-shaped polariton condensate above the threshold, where a pulse laser beam of the diameter of 1 μm is focused on the left side of the 10 μm ring to excite the sample. The red circle represents the position of the excitation laser.
